# Pharmacokinetics and efficacy of tank-water-administered dabrafenib, a BRAF inhibitor, in a zebrafish model of melanoma

**DOI:** 10.1242/dmm.052907

**Published:** 2026-10-16

**Authors:** Jenna Villman, Nikol Dibus, Jonas Huhn, Anna-Mari Haapanen-Saaristo, Ilia Evstafev, Alex M. Dickens, Oliver Scherf-Clavel, Ilkka Paatero, Kari J. Kurppa

**Affiliations:** ^1^ Turku Bioscience Centre, University of Turku and Åbo Akademi University, 20520 Turku, Finland; ^2^ Institute of Biomedicine and Medicity Research Laboratory, University of Turku, 20520 Turku, Finland; ^3^ Ludwig-Maximilians-Universität München, Department of Pharmacy, 81377 Munich, Germany

**Keywords:** Zebrafish, Melanoma, BRAF, Dabrafenib, Pharmacokinetics

## Abstract

Zebrafish models are widely used to study BRAF-mutant melanoma biology. However, long-term treatment of adult fish with small-molecule BRAF inhibitors remains challenging, and pharmacokinetic data to inform rational dosing strategies are largely lacking for most small molecules in zebrafish. Here, we assessed the pharmacokinetics, metabolism and efficacy of continuously tank-water-administered dabrafenib, a BRAF inhibitor, in adult zebrafish. Dabrafenib was rapidly absorbed from tank water, reaching efficacious plasma levels within 1 h, but also showed fast elimination kinetics with a half-life of 1.0 h. Most human metabolites of dabrafenib were detected in zebrafish, suggesting conserved metabolic processes. Continuous tank-water administration achieved therapeutically relevant steady-state dabrafenib plasma levels that inhibited BRAF-driven signaling and produced robust efficacy *in vivo* in a genetic zebrafish model of BRAF-mutant melanoma without apparent toxicity. Together, these results demonstrate that continuous tank-water administration of dabrafenib is a feasible, efficient and well-tolerated dosing strategy in zebrafish melanoma models, and may facilitate dosing of other small-molecule inhibitors, especially those with a short *in vivo* half-life in zebrafish.

## Teaser

**Summary:** Tank-water administration of dabrafenib is a feasible, efficient and well-tolerated dosing strategy in zebrafish models of melanoma and may facilitate dosing of other small molecules with short half-lives in zebrafish.

## INTRODUCTION

Melanoma incidence and deaths are constantly rising (Curti and Faries, 2021; Tsao et al., 2004). Although local melanoma can be surgically removed and has favorable prognosis, invasive or metastasized melanoma is typically highly aggressive, and the prognosis is poor (Tsao et al., 2004). In ∼50% of melanoma cases, the disease is driven by oncogenic mutations in the B-Raf proto-oncogene, serine/threonine kinase (*BRAF*) gene, most often by the BRAF V600E mutation (Curti and Faries, 2021). Patients with melanoma harboring BRAF mutations are clinically treated with BRAF-targeted therapy, such as dabrafenib or vemurafenib, often in combination with mitogen-activated protein kinase kinase 1/2 (MEK1/2; also known as MAP2K1/2) inhibitors (Curti and Faries, 2021). Although the response rates to BRAF-targeted therapy are typically very good, acquired resistance develops in almost all cases, preventing long-term benefit from therapy.

Zebrafish (*Danio rerio*) models have become important tools to understand melanoma genetics and cellular biology, especially in the context of BRAF-mutant melanoma. Since the introduction of zebrafish models of BRAF-mutant melanoma by Patton and others (Patton et al., 2005), numerous studies have used this model system to provide insight into melanoma development and progression. However, the long-term treatment of adult fish using BRAF-targeted therapy is challenging, limiting the usefulness of this model for studies on adaptive or intrinsic resistance mechanisms to BRAF-targeted therapy.

Previous studies have described the administration of the BRAF inhibitor vemurafenib to adult fish by oral gavage (Dang et al., 2016), or by pellets of vemurafenib-containing food (Lu and Patton, 2022). However, gavage to a fish of few centimeters in length is challenging and requires expertise, and may cause severe morbidity in the treated fish if done wrong. Also, continuous and even uptake of drug via food is difficult to control, potentially leading to uneven dosing. Furthermore, understanding of the pharmacokinetics of many small-molecule inhibitors, including ones targeting BRAF, in zebrafish is still limited and precludes rational dosing design.

Pharmacological and toxicological studies on larval zebrafish routinely administer drugs by supplementing the tank water with the study compounds. However, this route of administration is used to dose adult fish in e.g. acute toxicity studies (OECD, 2025) but less commonly used for longer efficacy studies. To date, it has not been used to dose small-molecule inhibitors in zebrafish models of melanoma. Here, we studied the feasibility, pharmacokinetics and efficacy of continuously tank-water-administered dabrafenib, a BRAF inhibitor, in adult fish, using a well-established zebrafish model of BRAF-mutant melanoma. Our results demonstrate that dabrafenib administered in the tank water is quickly absorbed into adult fish, reaching efficacious levels within 1 h of exposure to the drug. Furthermore, we demonstrate that dabrafenib is metabolized in zebrafish to the same metabolites as in humans and shows fast elimination kinetics. Treatment of tumor-bearing fish resulted in rapid and sustained tumor regression. Together, our results demonstrate that administering dabrafenib directly in tank water provides a feasible, efficient and well-tolerated method to study treatment-related effects in zebrafish models of BRAF-mutant melanoma.

## RESULTS

### Tank-water-administered dabrafenib has rapid pharmacokinetics in adult zebrafish and leads to sustained therapeutically relevant plasma levels over long-term treatment

To develop a cost-effective, fast and efficient method to dose adult zebrafish with the BRAF inhibitor dabrafenib over long treatment durations, we assessed the feasibility of directly administering the drug to the fish tank water. To analyze the pharmacokinetics of tank-water-administered dabrafenib in zebrafish, we exposed adult *mitfa(−/−),p53(−/−)* zebrafish to 10 μM dabrafenib [based on preliminary tolerability experiments (Fig. S1)] in tank water and collected post-mortem blood samples from fish exposed to dabrafenib for different times (Fig. 1A). These blood samples were then subjected to mass spectrometry (MS) analysis for dabrafenib and its active metabolite hydroxy-dabrafenib. Blood sampling was carried out after euthanasia and yielded 0.6-6.4 μl plasma per fish, with good correlation with fish weight (Fig. 1B). Despite low volumes, all samples were sufficient for MS analysis (Tables S1-S4). Based on the analysis, dabrafenib was absorbed rapidly and detected in zebrafish plasma after 1 h of exposure (Fig. 1C). Dabrafenib plasma levels peaked at 4 h post exposure, after which the levels started to decline, reaching 2.7±2.3 μM at 24 h post exposure, suggesting active metabolism of dabrafenib in zebrafish. Indeed, we were able to detect the dabrafenib metabolite hydroxy-dabrafenib soon after administration of dabrafenib, with similar kinetics to dabrafenib (Fig. 1D). To analyze the elimination of dabrafenib, we transferred zebrafish to fresh tank water after 24 h of dabrafenib exposure and monitored dabrafenib levels in fish plasma over time (Fig. 1A). Dabrafenib was cleared rapidly from zebrafish plasma with a half-life of 1.0 h (Fig. 1E), and similarly fast elimination kinetics (half-life, 1.6 h) were observed for hydroxy-dabrafenib (Fig. 1F). These results demonstrate fast absorption and elimination kinetics, as well as active metabolism of tank-water-administered dabrafenib in zebrafish, with therapeutically relevant (Puszkiel et al., 2019) steady-state plasma levels using 10 μM tank-water concentrations of dabrafenib. Indeed, after a 2-week treatment, the concentration of dabrafenib in fish plasma was 5.9±3.3 μM and that of hydroxy-dabrafenib was 0.14±0.12 μM (Table S1), indicating sustained therapeutically relevant levels over a long-term treatment period.

**Fig. 1.**
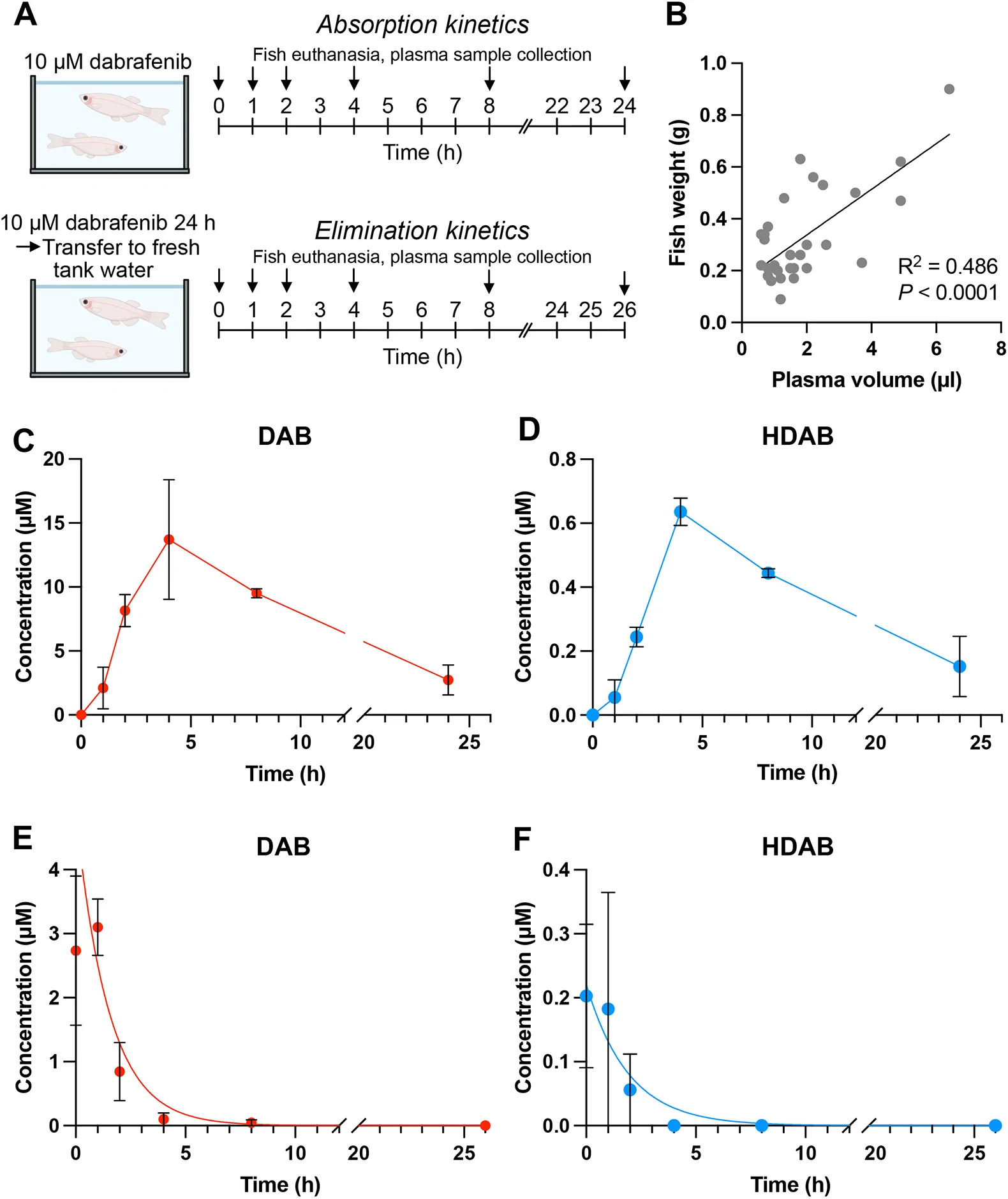
**Dabrafenib administered to tank water shows rapid absorption and elimination kinetics in zebrafish.** (A) Schematic overview of sample collection. To monitor absorption, the *mitfa(−/−),p53(−/−)* zebrafish were kept in tank water supplemented with 10 μM dabrafenib for different times before being culled, and blood samples were collected (*n*=2-4 for each timepoint). To monitor elimination, after 24-h drug exposure, the zebrafish were transferred to tank water without dabrafenib and culled at different time points (*n*=2-4 for each timepoint). (B) Terminally collected blood sample volumes correlated with fish weight. Pearson correlation. *N*=39. (C,D) Absorption of dabrafenib (DAB; C) and emergence of hydroxy-dabrafenib (HDAB; D) were measured from blood samples using mass spectrometry. (E,F) Elimination of dabrafenib (E) and hydroxy-dabrafenib (F) were measured from blood samples using mass spectrometry.

### Dabrafenib is metabolized in zebrafish through a similar metabolic process as in humans

To further analyze the metabolism and distribution of dabrafenib in zebrafish tissues, *mitfa(−/−),p53(−/−)* zebrafish exposed to 10 μM dabrafenib for 24 h were euthanized, snap frozen and cryosectioned. Cryosections were analyzed using MS imaging (Fig. 2A) for dabrafenib and its main metabolites in humans, hydroxy-dabrafenib, carboxy-dabrafenib and desmethyl-dabrafenib. In humans, dabrafenib is metabolized by cytochrome P450 (CYP) enzymes to hydroxy-dabrafenib, and further to carboxy-dabrafenib. Carboxy-dabrafenib can be excreted in bile and urine, or can undergo a non-enzymatic decarboxylation in the gut to desmethyl-dabrafenib (Puszkiel et al., 2019). We were able to detect dabrafenib, hydroxy-dabrafenib and desmethyl-dabrafenib in zebrafish tissues and very low levels of carboxy-dabrafenib (Fig. 2B), suggesting that dabrafenib undergoes similar metabolism in zebrafish as in humans. Although we cannot exclude the possibility of technical limitations in detecting carboxy-dabrafenib, it is possible that carboxy-dabrafenib undergoes fast non-enzymatic conversion to desmethyl-dabrafenib in zebrafish gut. Indeed, the localization of MS imaging signal was indicative of distribution of dabrafenib and its detected metabolites, especially in the gut (Fig. 2C), although tissue samples showed suboptimal integrity. Taken together, these observations suggest that dabrafenib is metabolized in zebrafish through a very similar metabolic process as in humans.

**Fig. 2.**
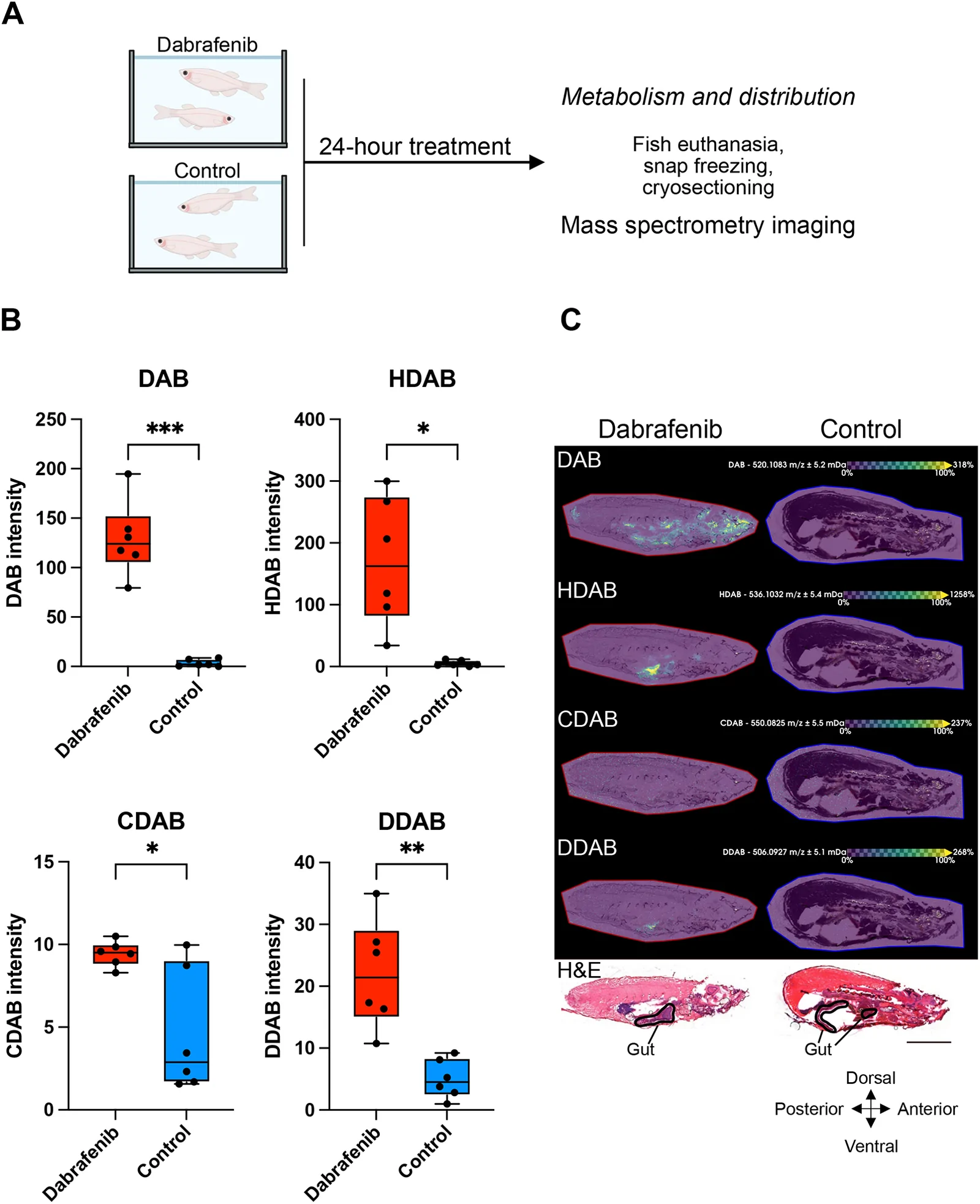
**Dabrafenib administered to tank water shows relevant metabolism and distribution in zebrafish.** (A) Schematic overview of sample collection. To monitor distribution, the *mitfa(−/−),p53(−/−)* zebrafish were kept in tank water supplemented with 10 μM dabrafenib for 24 h, euthanized and snap frozen. Frozen samples were cryosectioned sagittally and processed for mass spectrometry imaging. (B) The mean intensity of dabrafenib (DAB), hydroxy-dabrafenib (HDAB), carboxy-dabrafenib (CDAB) and desmethyl-dabrafenib (DDAB) in cryosections (*n*=6 for dabrafenib and control groups). Unpaired Welch's *t*-tests were used to assess statistical significance. *P<0.05; **P<0.01; ***P<0.001. In box-and-whisker plots, the box represents the 25-75th percentiles, and the median is indicated. Whiskers indicate minimum and maximum values. (C) Distribution of dabrafenib and its metabolites in zebrafish cryosections. Dabrafenib was widely distributed, whereas hydroxy-dabrafenib and desmethyl-dabrafenib were localized, presumably in the gut. Carboxy-dabrafenib levels were very low, and it could not be localized with sufficient confidence. Scale bar: 5 mm.

### Steady-state dabrafenib plasma levels are sufficient to inhibit mutant-BRAF-driven MAPK signaling *in vitro* and *in vivo*, and suppress proliferation of BRAF-driven zebrafish melanoma *in vitro*

Oncogenic mutations in BRAF, such as BRAF V600E, lead to hyperactivation of the downstream mitogen-activated protein kinase (MAPK) pathway, which fuels the growth of melanoma cells. In order to assess whether the reached steady-state dabrafenib plasma concentrations were sufficient to suppress MAPK signaling and proliferation of BRAF V600E-driven zebrafish melanoma, we analyzed ZMEL1 cells (Heilmann et al., 2015) *in vitro*. A western blot analysis of phospho-ERK (also known as phospho-MAPK) levels indicated that dabrafenib effectively inhibits MAPK signaling in ZMEL1 cells, with a half-maximal inhibitory concentration (IC50) of 88 nM (95% CI 43-186 nM), without affecting total ERK protein levels [extra sum-of-squares *F*-test, *F* (DFn, DFd)=149.5 (3,48), *P*<0.0001] (Fig. 3A,B). Consistently, we observed a dose-dependent reduction in ZMEL1 cell growth with an IC50 of 74 nM (95% CI 57-97 nM) (Fig. 3C). Both IC50 values were well below the reached steady-state dabrafenib plasma levels of 2.7 μM. Indeed, this concentration led to more than 80% reduction in phospho-ERK levels and cell proliferation in ZMEL1 cells (Fig. 3B,C). To assess the effect of tank-water-administered dabrafenib on inhibiting MAPK signaling *in vivo*, we performed multiplexed immunohistochemistry on tissue sections from tumor-bearing *mitfa(−/−),p53(−/−), Tg(mitfa:BRAF(V600E))* zebrafish treated with dabrafenib or DMSO for 2 weeks. We observed a significant reduction in phospho-ERK signal in BRAF V600E-positive tumor areas in fish treated with dabrafenib, compared to that in DMSO-treated controls (Fig. 3D,E). Taken together, these results indicated that tank-water-administered dabrafenib results in steady-state dabrafenib plasma levels that inhibit MAPK signaling in BRAF V600E-driven zebrafish melanoma *in vitro* and *in vivo*, and suppress zebrafish melanoma growth *in vitro*.

**Fig. 3.**
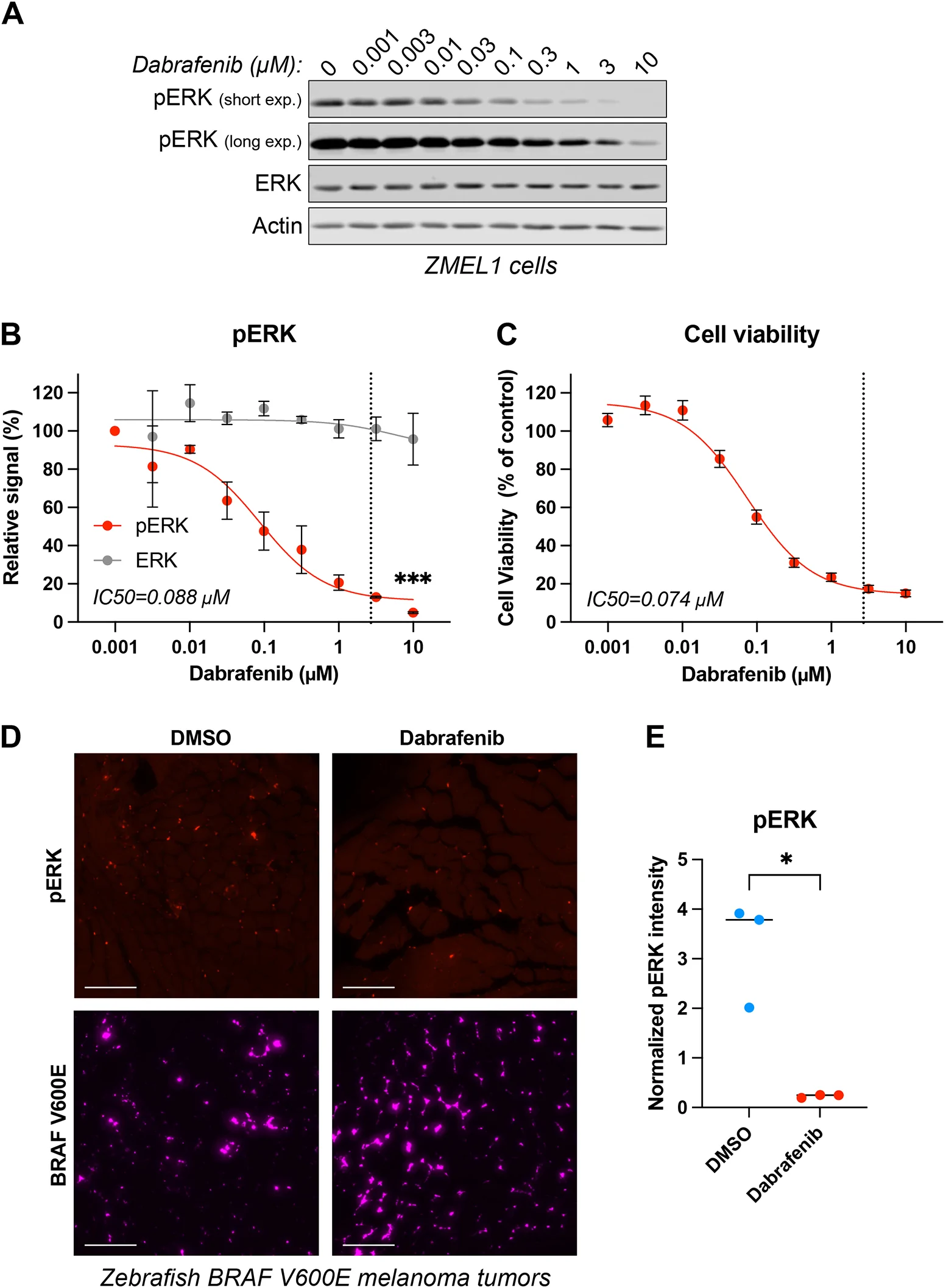
**Steady-state dabrafenib plasma levels are sufficient to inhibit mutant BRAF-driven MAPK signaling *in vitro* and *in vivo*, and suppress proliferation of BRAF-driven zebrafish melanoma *in vitro*.** (A) Dabrafenib-induced reduction in MAPK activity in BRAF-driven zebrafish melanoma cells. ZMEL1 cells were treated for 24 h with the indicated concentrations of dabrafenib, and protein lysates were analyzed for phospho-ERK using western blotting. (B) Quantification of the results in A. The data represent the average of three independent experiments and are shown as mean±s.d. Extra sum-of-squares *F*-test was used to assess the difference between dabrafenib dose–response curves for ERK and phospho-ERK. ***P<0.001. (C) Dabrafenib-induced reduction in ZMEL1 cell proliferation. The cells were treated with the same dabrafenib concentrations as in A for 72 h, then viability was measured. The data shown represent the average of three independent experiments and are shown as mean±s.e.m. The steady-state dabrafenib plasma concentration shown in Fig. 1C is indicated as a vertical dashed line in B and C. IC50, half-maximal inhibitory concentration. (D) Multiplexed immunohistochemistry assessing phospho-ERK in melanoma tumors of *mitfa(−/−),p53(−/−), Tg(mitfa:BRAF(V600E))* zebrafish treated with DMSO or dabrafenib for 2 weeks (*n*=3 for both groups). A BRAF V600E-specific antibody was used to detect melanoma cells. Representative images are shown. Scale bars: 100 μm. (E) Quantification of phospho-ERK signal in D. Phospho-ERK signal was normalized to BRAF V600E signal in each image. Unpaired Welch's *t*-test was used to assess statistical significance. *P<0.05.

### Tank-water-administered dabrafenib results in rapid and sustained tumor regression in a zebrafish model of BRAF-mutant melanoma

To assess the *in vivo* efficacy of tank-water-administered dabrafenib in a genetic zebrafish model of BRAF-mutant melanoma, we treated melanoma tumor-bearing *mitfa(−/−),p53(−/−), Tg(mitfa:BRAF(V600E))* zebrafish for 2 weeks with 10 μM dabrafenib. Dabrafenib was administered into tank water, the fish were weighed and imaged with a fixed mobile phone, and the dosing was replenished every 3-4 days (Fig. 4A). The tumor area in each timepoint was quantified from the images using QuPath software, and the change in tumor area compared to baseline was calculated. Consistent with our *in vitro* analyses, the tank-water-administered dabrafenib demonstrated robust efficacy *in vivo*, with nine of 13 (69%) skin melanoma tumors responding to treatment (Fig. 4B), which is in line with clinical studies with BRAF inhibitors in human patients with BRAF-mutant melanoma (Chapman et al., 2011; Hauschild et al., 2012). All of the nine responding tumors regressed more than 50% in size, with near complete responses in three cases. Overall, tank-water-administered dabrafenib resulted in significant reduction in skin melanoma tumor burden in the treated fish, compared to that in DMSO-treated controls (Fig. 4C). In some fish, we observed spontaneous development of pigmented tumors in the eye, with an incidence of 32% (nine of 28 fish) (Fig. 4D). Although the sample size was small, we observed a reduction in tumor size of more than 30% in all of the eye tumors treated with dabrafenib (Fig. 4B,C), suggesting that these tumors are also BRAF driven. Consistent with the rapid absorption and sufficient steady-state levels of tank-water-administered dabrafenib (Fig. 1), the treatment resulted in fast and sustained regression of the responding tumors in skin melanomas and eye tumors (Fig. 4E). Importantly, tank-water-administered dabrafenib was well tolerated, with no significant weight loss (Fig. 4F) or mortality during the 2-week treatment. Taken together, these results indicated that tank-water-administered dabrafenib is well tolerated in adult zebrafish over long treatment duration, and results in rapid and sustained tumor regression of BRAF-driven zebrafish melanoma *in vivo*.

**Fig. 4.**
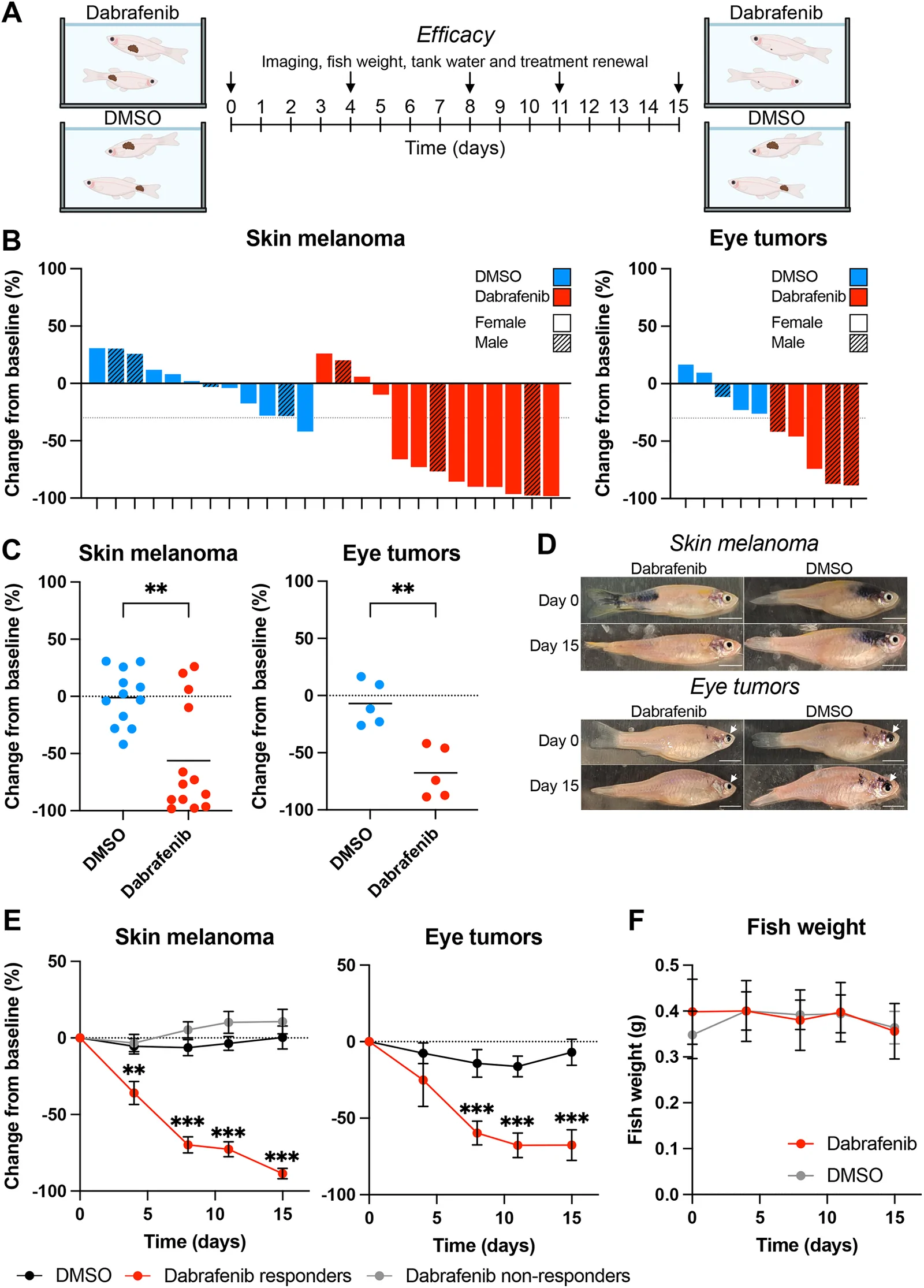
**Tank-water-administered dabrafenib results in rapid and sustained tumor regression in a zebrafish model of BRAF-mutant melanoma.** (A) Schematic presentation of the efficacy experiments. Tumor-bearing *mitfa(−/−),p53(−/−), Tg(mitfa:BRAF(V600E))* fish were treated with 10 μM dabrafenib or DMSO administered in the tank water. The fish were weighed and imaged, and the dosing was replenished every 3-4 days. The tumor areas of all individual tumors in the study fish were determined from images from all timepoints (see Materials and Methods). (B) Waterfall plots demonstrating the skin (left) or eye (right) tumor area change from baseline after 15 days of treatment for each individual tumor in the study fish (*n*=22 fish with 25 skin tumors and ten eye tumors). 30% reduction in tumor area is shown as a horizontal dotted line. (C) Scatter plot demonstrating the overall skin (left) or eye (right) tumor area change from baseline for all tumors in the DMSO or dabrafenib cohorts. (D) Representative images from DMSO- or dabrafenib-treated fish bearing skin melanoma (top) or an eye tumor (bottom). Scale bars: 5 mm. (E) Skin (left) or eye (right) tumor area change from baseline over time in the DMSO cohort, as well as in the dabrafenib cohort responding or not responding to treatment. Data are shown as mean±s.e.m. (F) Fish weight over time in DMSO- or dabrafenib-treated cohorts. Individual unpaired Welch's *t*-tests (C) or multiple unpaired Welch's *t*-tests with multiple comparisons testing using two-stage step-up method (E) were used to evaluate statistical significance. Asterisks indicate *P*-values (C) or *q*-values (E): **P<0.01; ***P<0.001.

## DISCUSSION

In this work, we studied the suitability of tank-water administration of the BRAF inhibitor dabrafenib for long-term treatment studies in adult zebrafish. We tested the absorption, distribution, excretion and metabolism of tank-water-administered dabrafenib in adult fish, as well as the treatment efficacy in a genetic zebrafish model of BRAF-mutant melanoma.

Dabrafenib administered to tank water was rapidly absorbed by zebrafish. Plasma levels exceeding *in vitro* IC50 values were reached within 1 h of treatment and maintained over the course of the 2-week experiment. Dabrafenib also demonstrated fast elimination kinetics, with a plasma half-life of 1.0 h, suggesting rapid metabolism in zebrafish. Indeed, we detected most of the known human metabolites of dabrafenib in zebrafish, suggesting that the metabolism of dabrafenib follows a similar metabolic process in zebrafish as it does in humans. Consistent with the fast absorption kinetics and therapeutically relevant steady-state plasma levels, tank-water-administered dabrafenib led to rapid and sustained tumor regression in a genetic zebrafish model of BRAF-mutant melanoma. We observed responses in melanomas of the skin as well as in spontaneously occurring pigmented tumors in the eye. Intriguingly, although human uveal melanomas of the eye almost exclusively lack BRAF mutations, a rarer conjunctival subtype of ocular melanoma has been described to harbor frequent BRAF mutations (Griewank et al., 2013). Future studies should characterize these zebrafish eye tumors in detail and assess their potential similarity to human ocular melanomas. In skin melanomas, the treatment resulted in similar response rates as observed for human patients with BRAF-mutant melanoma treated with BRAF inhibitors (Chapman et al., 2011; Hauschild et al., 2012). Interestingly, clearly intrinsically resistant tumors were also observed. In this study, we did not assess the potential long-lasting effects of the dabrafenib treatment by measuring tumor regrowth following treatment cessation. Considering the fast elimination kinetics of dabrafenib, we would expect tumor regrowth to occur in most cases, at least those with measurable residual tumor burden. In addition, we did not treat the fish beyond 2 weeks and, thus, are unable to evaluate whether continued treatment would lead to the development of resistance as frequently observed in patients and previously also in zebrafish models (Lu and Patton, 2022), although we find it likely.

Previous reports have described administration of small-molecule BRAF inhibitors to zebrafish using gavage (Dang et al., 2016) or feeding (Lu and Patton, 2022). Repeated gavage is a highly technical procedure, requires skilled personnel and may cause severe morbidity in the treated fish. Both gavage and feeding can be administered only once or a couple of times per day, whereas administration to tank water provides infusion-like continuous administration. A related BRAF inhibitor, vemurafenib, has been administered in pelleted food once a day, with a clear therapeutic response (Lu and Patton, 2022). The pharmacokinetics of vemurafenib were not determined in this study; but, at least in humans, vemurafenib has a significantly longer half-life (Flaherty et al., 2010; Grippo et al., 2014) than dabrafenib (Falchook et al., 2012). The drug administration via food facilitates feasible long-term experiments; however, in the case of drugs with a short *in vivo* half-life in zebrafish (such as dabrafenib), administration into tank water may better facilitate the maintenance of effective therapeutic doses throughout long-term treatment. In this regard, repeated administration through injection (Kinkel et al., 2010), oral gavage (Dang et al., 2016) or short daily baths (Monstad-Rios et al., 2018) in drug solutions may have similar limitations. Administration directly into tank water is a technically easier and well-tolerated method, but the largest drawback is the relatively large amount of drug needed to treat the fish. The tank-water-administration method could be further improved to include monitoring of drug levels in water or utilization of a continuous supply of drug using automated dosing and flow-through systems to maintain very stable drug exposure levels and automated, continuous water changes for optimal water quality during the experiment. These setups have been already developed and utilized in toxicological experiments (Ensenbach and Nagel, 1997).

Pharmacokinetics is typically understudied in zebrafish, and head-to-head comparisons of different delivery routes are largely lacking. The comparison of kinetics using different administration routes would be useful to enable rational design of most optimal drug administration methods for experiments. It is unlikely that any single administration method would be suitable for all cases, and rational planning of administration should be part of the experimental design as is routine e.g. in rodent studies. In all administration methods, the analysis of drug levels in blood and/or tissues enables confirmation of effective drug administration. This can be further enhanced by analysis of drug kinetics in pilot experiments to facilitate rational planning of dosing for larger treatment experiments. For future studies, systematic analysis of pharmacokinetics would be warranted, as zebrafish models are increasingly used in drug discovery and development projects, in addition to cancer studies.

To conclude, tank-water administration is a feasible, efficient and well-tolerated method for treatment of adult zebrafish with dabrafenib, facilitating long-term studies on treatment-induced effects in BRAF-driven tumor biology. We anticipate that this method also extends to other small-molecule inhibitors, especially in the case of molecules with short *in vivo* half-lives in zebrafish.

## MATERIALS AND METHODS

### Zebrafish husbandry

Zebrafish of the *mitfa(−/−),p53(−/−)* strain (*mitfa*, *w2* allele; *p53*, *zdf1* allele) (Berghmans et al., 2005; Lister et al., 1999) (a kind gift from Adam Hurlstone, University of Manchester, Manchester, UK) were maintained in accordance with standard protocols (Westerfield, 2000) under fresh water flow, and fed with Gemma micro 300 dry food twice daily on weekdays and once daily on weekends. All experiments were conducted under a license obtained from the Animal Experimental Board of the Regional State Administrative Agency for Southern Finland (ESAVI/2688/2020).

### Tank-water administration of dabrafenib

Adult 5- to 20-month-old *mitfa(−/−),p53(−/−)* or *mitfa(−/−),p53(−/−), Tg(mitfa:BRAF(V600E))* zebrafish (two to three fish in a 2-l tank) were treated with 10 μM dabrafenib (HY-14660, MedChemExpress) by diluting dabrafenib directly into the tank water from a 50 mM (in DMSO) stock solution at the start of the treatment. An equivalent volume of DMSO was added to the tank water of control fish. The treated fish were maintained in static conditions without continuous water flow. In short 24-h experiments, the fish were fed either directly before (absorption kinetics) and after (elimination kinetics) drug exposure. In long-term experiments, the fish were fed once daily, and the tank water and dabrafenib were replenished every 3-4 days by replacing half of the water volume with fresh water containing 10 μM dabrafenib. During tank water and dosing replenishment, the fish were imaged and weighed under light anesthesia with 160 mg/l tricaine (Cas:886-86-2, Sigma-Aldrich), allowed to recover in a tank without dabrafenib and placed back into the treatment tank. The total duration of the procedure was ∼5-10 min. Oxygen and ammonium (NH_4_) levels were measured every third day to monitor water quality. The 2-l experiment tanks were not cleaned during the experiments.

### Pharmacokinetic analysis of plasma samples

For determining the absorption kinetics of dabrafenib, blood samples were taken from the fish at different time points after drug administration (0, 1, 2, 4, 8, 24 h and 2 weeks). To determine elimination kinetics, fish were treated for 24 h with 10 μM dabrafenib, then fed and transferred to a new 2-l tank with fresh tank water. Blood samples were taken from the fish at different timepoints after transfer to fresh tank water (0, 1, 2, 4, 8, 26 h). Blood samples were collected by transecting the tail between caudal and anal fin with a steel blade (Swann-Morton) after chemical euthanasia with 400 mg/l tricaine. The tubes and pipette tips were coated with 0.5 M EDTA (pH 8.5, Cas:6381-92-6, VWR Chemicals) in milliQ water and air dried. The samples were centrifuged at room temperature, 2000 ***g*** for 5 min with a bench-top centrifuge. The plasma was moved to a new EDTA-coated tube. Samples were snap frozen on dry ice and stored at −80°C.

### Quantification of dabrafenib and hydroxy-dabrafenib by liquid chromatography–tandem mass spectrometry (LC-MS/MS)

#### Standards and calibration matrix

Blank human plasma was obtained from Blutspendedienst des Bayerischen Roten Kreuzes gGmbH (Munich, Germany). Dabrafenib mesylate (referred to as dabrafenib) was purchased from Sigma-Aldrich (Taufkirchen, Germany). Dabrafenib-d9 (internal standard) was purchased from Alsachim (Illkirch Graffenstaden, France), and hydroxy-dabrafenib was purchased from Biozol Diagnostica (Hamburg, Germany).

#### Stock solutions and working solutions

Dabrafenib, hydroxy-dabrafenib and dabrafenib-d9 stock solutions were prepared in DMSO to yield a concentration of 1 mg/ml. Two independent stock solutions were used for the preparation of calibration standards and quality control (QC) samples. All solutions were stored at −80°C. Dabrafenib and hydroxy-dabrafenib were combined in a working stock solution (solvent: methanol) to yield concentrations of ∼30 μg/ml for dabrafenib and hydroxy-dabrafenib. Dabrafenib-d9 was diluted in DMSO to 20 μg/ml to obtain an internal standard (IS) working stock solution. The IS working stock solution was diluted with acetonitrile (ACN) to yield the precipitating agent at an IS concentration of ∼100 ng/ml dabrafenib-d9. All concentrations refer to the concentration of free base.

#### Calibration solutions and QC samples

The calibration curve in matrix was prepared by spiking 950 μl blank human plasma with 50 μl working stock solution to obtain the highest calibration standard. For the highest QC sample, 960 μl blank human plasma was spiked with 40 μl QC working stock solution. The remaining QC samples and calibrators were obtained by serial dilution with blank human plasma. The calibration range for dabrafenib and hydroxy-dabrafenib was 5-1500 ng/ml. QC high, QC middle and QC low were at a concentration of 1200, 600 and 15 ng/ml, respectively. A weighted calibration (1/concentration^2^) was applied for both analytes.

#### Sample preparation

As the samples contained less than 25 μl plasma, the available volume was adjusted to 25 μl with blank human plasma. 75 μl ACN containing IS was added to 25 μl sample, calibrator or QC sample. After vortexing for 15 s, the samples were centrifuged for 5 min at 4°C and 11,000 ***g***. 50 μl supernatant was transferred to another tube containing 450 μl of a mobile phase mixture (mobile phase A/mobile phase B 60:40, v/v) and vortexed for another 10 s. 300 μl was transferred into a deep-well plate and injected into the high-performance liquid chromatography (HPLC) system.

#### LC-MS/MS experiment

The samples were measured on an API3200 (Sciex, Darmstadt, Germany) combined with an Agilent 1200 HPLC (Agilent, Waldbronn, Germany). The separation was carried out on a Waters XBridge Phenyl column 2.1×50 mm (3.5 μm particle size). Mobile phase A consisted of methanol/water (1:9, v/v) containing 10 mM NH_4_HCO_3_, whereas mobile phase B was methanol/water (9:1, v/v) containing 10 mM NH_4_HCO_3_. The separation was carried out in gradient separation mode at a flow rate of 400 μl/min. The injection volume was 5 μl. The gradient program was as follows: 40% A/60% B held for 0.5 min, followed by a linear decrease to 20% A/80% B over 1.5 min. This composition was maintained isocratically for 3 min, after which the mobile phase was returned to the initial conditions (40% A/60% B) within 0.25 min and held for 1.75 min to allow column re-equilibration. The total run time was 7 min. Analytes were monitored in positive electrospray ionization (ESI+) mode using multiple reaction monitoring. For dabrafenib, the transition 520.0→307.1 m/z was used with a declustering potential (DP) of 81 V and a collision energy (CE) of 43 V. Hydroxy-dabrafenib was monitored using the transition 536.0→323.2 m/z with identical source parameters (DP, 81 V; CE, 43 V). The internal standard dabrafenib-d9 was monitored using the transition 529.0→316.0 m/z (also with DP, 81 V; CE, 43 V). The source parameters were as follows: Turbo IonSpray source (Sciex) at 400°C and 5500 V using source gas 1 and gas 2 at 30 units, curtain gas at 25 units and collisionally activated dissociation gas at 6 units.

### MS imaging

Zebrafish treated with 10 μM dabrafenib for 24 h were snap frozen in a dry-ice bath with isopropanol and moved into −80°C. One fish at a time was embedded on its left side on a specimen disc with M-1 Embedding Matrix (1310, Epredia) inside a cooled Leica CM1950 or Leica CM3050 cryostat, and cut into 15-16 μm sections. The MS imaging runs were performed by the Turku Metabolomics Core Facility. Six dabrafenib-treated samples and six control samples were processed. The matrix was applied using the sublimation method. Super-DHB (Sigma-Aldrich) at a concentration of 40 mg/ml dissolved in acetone was used as a matrix for matrix-assisted laser desorption/ionization. A vacuum was created using an MD 1C+AK+EK chemistry system (VACUUBRAND GmbH, Wertheim-Bestenheid, Germany). The matrix solution (750 μl) was applied to the bottom of the sublimator chamber and dried under a nitrogen flow, after which a slide with a sample was installed and a vacuum was created. Sublimation was carried out at a temperature of 140°C for 15 min.

Samples were measured by Bruker timsTOF fleX equipped with postionization laser (Bruker Daltonics) in positive mode from 300 to 1350 m/z with 100 lasershots per pixel using 50 μm spot size settings (50 μm spot size; 50 μm step size). All imaging experiments were loaded into SCiLS Lab (v2023bPro, Bruker Daltonics) using the default settings with the parameters from FlexImaging. Data were searched for following targets with 10 ppm error window: dabrafenib (520.108 m/z), carboxy-dabrafenib (550.083 m/z), desmethyl-dabrafenib (506.093 m/z), hydroxy-dabrafenib (536.103 m/z). The data were not normalized owing to scarce distribution of selected targets in analyzed samples. The intensity data were exported to .csv files and analyzed in GraphPad Prism.

### Hematoxylin–Eosin staining of MS slides

After MS imaging, the matrix was removed from samples with 15 min incubation in acetone (Cas:67-64-1, Sigma-Aldrich). Samples were incubated twice in 100% ethanol for 3 min, once in 70% ethanol for 3 min and once in milliQ water for 2 min. Slides were placed in Mayer's Hematoxylin solution (MHS16, Sigma-Aldrich) for 10 min, then rinsed for 10 min under running tap water, followed by 30 s incubation in milliQ water and 30 s incubation in 96% ethanol. Then, slides were stained with alcoholic Eosin Y solution (HT110116, Sigma-Aldrich) for 45 s, followed by a 1 min wash in 96% ethanol and two 3 min washes in 100% ethanol. The slides were then soaked twice in xylene for 5 min, mounted with DPX mountant (06522, Sigma-Aldrich) and covered with a coverslip. The slides were scanned with a Pannoramic P1000 (3DHISTECH) slide scanner by a 40×/0.95 NA objective and analyzed using QuPath (0.3.2).

### Cell culture

The ZMEL1-GFP (generously provided by Dr Richard White, Memorial Sloan Kettering Cancer Center, New York, NY, USA) cells were cultured in DMEM High Glucose medium (ECB7501L, Euroclone) containing 10% fetal bovine serum (FBS; Biowest), 200 mM glutamine (Euroclone) and 100 U/ml penicillin/100 μg/ml streptomycin (Euroclone). The cells were incubated in a humidified incubator at 28°C with 5% CO_2_. ZMEL1 cells were maintained at low passage and were not authenticated. Cells were tested for mycoplasma infection using MycoAlert (Lonza).

### Dose–response assay

The ZMEL1-GFP cells were seeded in a 96-well plate at a density of 5000 cells/well in 100 μl culture medium and allowed to adhere for 24 h. Dabrafenib with concentrations ranging from 1 nM to 10 μM was added to the cells in triplicates using an automatic HD D300 Digital Dispenser (HP). After 72 h incubation, 30 μl CellTiter-Glo Luminescent Cell Viability Assay Solution (Promega) was added to each well, and the plate was incubated on a shaker (Certomat MO, Braun Melsungen AG) at room temperature for 10 min in dark. Luminescence was measured using an Agilent Biotek Cytation5 cell imaging multimode reader (AH diagnostics).

### Western blotting

The ZMEL1-GFP cells (5×10^6^ cells) were seeded on 10 cm dishes. After 24 h, the cells were treated with dabrafenib concentrations ranging from 1 nM to 10 μM and incubated for a further 24 h. Following treatment, cells were collected in PBS by scraping. After pelleting, the cells were lysed in 150 μl lysis buffer (150 mM NaCl, 50 mM Tris-HCl pH 7.5, 0.4% Triton X-100, 2 mM CaCl_2_, 2 mM MgCl_2_, 1 mM EDTA, 5 mM NaF in milliQ water) supplemented with Halt Protease and Phosphatase Inhibitor Cocktail (Thermo Fisher Scientific) and Genius Nuclease (Santa Cruz Biotechnology) on ice for 30 min. Protein concentration was determined using Pierce BCA Protein Assay (Thermo Fisher Scientific) according to the manufacturer's protocol. 30 μg total protein sample was mixed with 6× Laemmli buffer and heated at 95°C for 5 min. Proteins were separated by SDS-PAGE and transferred onto a nitrocellulose membrane. The membranes were washed with milliQ water and blocked with 5% fat-free milk in TBST (150 mM NaCl, 0.005% Tween-20, 10 mM Tris-HCl pH 7.5 in milliQ water) for 30 min at room temperature.

Membranes were incubated overnight at 4°C with anti-phospho-p44/42 MAPK (Thr202/Tyr204) antibody (1:1000, 9101, Cell Signaling Technology, RRID:AB_331646) or anti-p44/42 MAPK antibody (1:1000, 9102, Cell Signaling Technology, RRID:AB_330744) diluted in 5% bovine serum albumin in TBST with 0.01% NaN_3_. After washing with TBST, membranes were incubated with IRDye 800CW Goat or Donkey anti-Rabbit (1:10 000, LI-COR) diluted in blocking buffer for 1.5 h at room temperature. After further TBST washes, signal detection was performed using an Odyssey CLx Imager (LI-COR). To control for protein loading, the membranes were stripped with stripping buffer (25 mM glycine-HCl, 1% SDS pH 2) for 20 min and blocked with 5% fat-free milk in TBST before incubating with monoclonal anti-β-actin antibody (1:1000, A2228, Sigma-Aldrich, RRID:AB_476697) and IRDye 680RD Donkey anti-Mouse (1:10,000, LI-COR). The western blot images were quantified using ImageStudio software (version 5.5), and phospho-ERK and ERK band intensities were normalized to the actin signal in each lane.

### Establishment of melanoma tumors in zebrafish

Adult *mitfa(−/−),p53(−/−)* zebrafish were bred, and the embryos were collected next morning. Embryos were injected at one-cell stage with an injection volume of 2.3 nl of 12.5 ng/μl miniCoopR BRAF-V600E-2A-EGFP plasmid, 12.5 ng/μl Tol2TP RNA, 150 mM KCl and Phenol Red in ultrapure water. Injections were done using Nanoject II (3-000-205A, Drummond) injector and glass needles pulled from glass capillaries (TW100-4, World Precision Instruments Ltd., Sarasota, FL, USA) using a micropipette puller (PB-7, Narishige, Tokyo, Japan). After microinjection, embryos were maintained in E3 medium (5 mM NaCl, 0.17 mM KCl, 0.33 mM CaCl_2_, 0.33 mM MgSO_4_) with penicillin/streptomycin at 28.5°C. At 1 day post injection (dpi), 20 mg/ml pronase E (Cas:9036-06-0, Merck) solution was added to embryos. At 3 dpi, embryos were selected based on patches or individual dark melanocytes using a Leica MZ6 stereomicroscope (Leica Microsystems). A total of 28 fish developed tumors, with 25 fish harboring skin tumor(s) and nine fish harboring pigmented tumor(s) in the eye. Three fish had only eye tumors.

### Multiplexed immunohistochemistry

Tumor-bearing *mitfa(−/−),p53(−/−), Tg(mitfa:BRAF(V600E))* zebrafish treated with DMSO or 10 μM dabrafenib for 2 weeks were fixed in 10% neutral buffered formalin, decalcified, dehydrated through ascending alcohol series, cleared with xylene, embedded into paraffin and sectioned at 4 μm thickness. Tissue section slides were baked for 30 min at +60°C, deparaffinized by xylene, and rehydrated in graded ethanol and water. Heat-induced epitope retrieval was performed in 2100 Retriever (Aptum Biologics, Ltd) in Tris-EDTA (pH 9), followed by endogenous peroxidase blocking by using 3% hydrogen peroxide for 10 min. Two staining rounds were carried out as follows: 5 min protein blocking in BrightDiluent diluent (BD09-999-IL, Immunologic), 20 min primary antibody incubation [1:800 Phospho-p44/42 MAPK (Thr202/Tyr204), Cell Signaling Technology, RRID:AB_2315112 and 1:100 BRAF V600E, Abcam, RRID:AB_2861282], 10 min secondary antibody incubation (BrightVision 1 step detection system goat anti-rabbit HRP and goat anti-mouse HRP, DVPM110HRP-IL/DVPR110HRP-IL, Immunologic), tyramide signal amplification (TSA) by incubating slides with fluorophore-conjugated tyramides (CF594 and CF640R CF Dye Tyramides, 92175/92174, Biotium) for 10 min in 4 μM final concentration in TBST, supplemented with fresh 0.0015% hydrogen peroxide. TSA reaction was quenched with 3% hydrogen peroxide (10 min) between staining cycles. Slides were washed between steps by TBST and mounted with ProLong Gold Antifade Mountant with DAPI (P36935, Thermo Fisher Scientific). Normal antibody diluent was used as a negative control instead of primary antibodies.

The stained slides were imaged using a Zeiss Axioscan 7 slide scanner at 20× magnification, with 385 nm, 585 nm and 635 nm channels acquired via a Kromnigon SpectraSplit 7 filter. The slides were analyzed using QuPath (0.3.2) software. The tumor regions were identified by BRAF V600E mutation-positive tumor cells from the 647 nm channel, and the phospho-ERK intensity of the tumor region was measured from the 590 nm channel. The tumor phospho-ERK intensity was normalized by dividing the phospho-ERK signal by the BRAF V600E signal in each image.

### Efficacy studies and tumor size measurement

Tumor-bearing *mitfa(−/−),p53(−/−), Tg(mitfa:BRAF(V600E))* fish of both sexes were housed in groups of two to three fish. DMSO or 10 μM dabrafenib was administered into the tank water as above. Three studies at separate times were performed (total *n*=22 fish with 25 skin tumors and ten eye tumors). The fish were visually inspected and fed daily, and ammonium and oxygen levels were measured every third day to monitor water quality. The fish were weighed and imaged on days 0, 4, 8, 11 and 15 during the experiment under anesthesia with 160 mg/l tricaine. The tank water and the dabrafenib were replenished as above at the same time points. Images were taken from both sides of each fish with a mobile phone attached to a stand at a fixed height. Every image included a ruler as a scale bar, which was used to normalize the area measurements. The images were analyzed using QuPath (0.3.2) and quantified by outlining the area of the pigmented tumors based on pixel intensities using the wand tool. Percentage change for matching pre- and post-treatment lesions was calculated based on the tumor areas. Response was determined to be over 30% decrease from baseline.

### Statistical methods

The statistical analyses were performed, and graphs were generated, using GraphPad Prism 10.4.2. Data are presented as mean±s.d. or s.e.m. For comparisons between two groups, unpaired two-tailed *t*-tests were used. Details of the tests used are found in figure legends. For comparing dose–response curves, extra sum-of-squares *F*-test was used. Comparisons with *P*-values less than 0.05 were considered statistically significant.

## Supplementary Material

10.1242/dmm.052907_sup1 Supplementary information

Table S1. Dabrafenib and hydroxy-dabrafenib concentrations in zebrafish plasma samples.

Table S2. MS run details.

Table S3. MS raw data – dabrafenib.

Table S4. MS raw data – hydroxy-dabrafenib.
