# Supplementary material for: Pharmacokinetics and efficacy of tank-water-administered dabrafenib, a BRAF inhibitor, in a zebrafish model of melanoma
Source: Dis Model Mech. 2026 Oct 16;19(10):dmm052907. doi: 10.1242/dmm.052907 (PMC13714868; doi:10.1242/dmm.052907)
Supplement: Supplementary file 1 [file dmm-19-052907-s1.pdf]

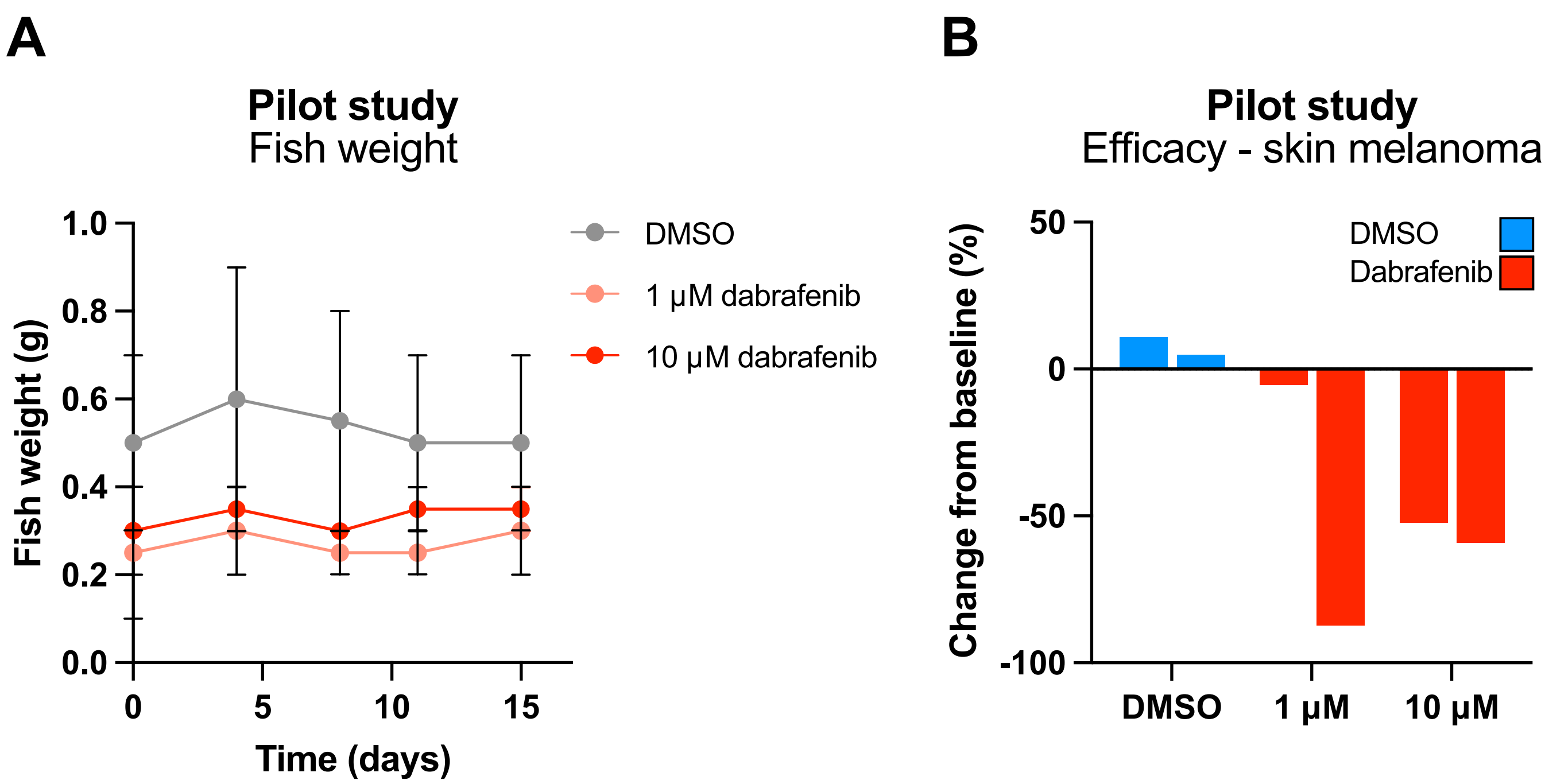

Fig. S1. Pilot study to assess the tolerability of tank water-administered dabrafenib. Tumor-bearing *mitfa(-/-),p53(-/-), Tg(mitfa:BRAF(V600E))* fish were treated with 1 or 10 µM dabrafenib or DMSO administered in the tank water. The fish were monitored daily, and the fish were weighed, imaged and the dosing replenished every 3-4 days. **A)** Fish weight over time in DMSO- or dabrafenib treated groups (n = 2 fish / group). **B)** The tumor areas of the tumors in the study fish were determined from images taken after two weeks of treatment (see methods for details). None of the fish in either of the dosing groups showed signs of toxicity.

Table S1. Dabrafenib and hydroxy-dabrafenib concentrations in zebrafish plasma samples.

Available for download at
https://journals.biologists.com/dmm/article-lookup/doi/10.1242/dmm.052907#supplementary-data

Table S2. MS run details.

Available for download at
https://journals.biologists.com/dmm/article-lookup/doi/10.1242/dmm.052907#supplementary-data

Table S3. MS raw data – dabrafenib.

Available for download at
https://journals.biologists.com/dmm/article-lookup/doi/10.1242/dmm.052907#supplementary-data

Table S4. MS raw data – hydroxy-dabrafenib.

Available for download at
https://journals.biologists.com/dmm/article-lookup/doi/10.1242/dmm.052907#supplementary-data
